# New insights into YAP/TAZ nucleo‐cytoplasmic shuttling: new cancer therapeutic opportunities?

**DOI:** 10.1002/1878-0261.12498

**Published:** 2019-05-17

**Authors:** Michal Shreberk‐Shaked, Moshe Oren

**Affiliations:** ^1^ Department of Molecular Cell Biology Weizmann Institute of Science Rehovot Israel

**Keywords:** exportin 1, hippo pathway, nucleo‐cytoplasmic shuttling, TAZ, YAP

## Abstract

Yes‐associated protein (YAP) and transcriptional co‐activator with PDZ‐binding motif (TAZ), the main effectors of the Hippo pathway, are emerging as important players in cancer biology and therapy response. The intracellular localization of YAP/TAZ is a key determinant in the regulation of their activity and their roles in signal transduction. This is particularly relevant for cancer: Aberrant nuclear localization of YAP and TAZ has been observed in numerous human cancers and may therefore represent an attractive target for cancer therapy. In this review, we describe the mechanisms that regulate the nucleo‐cytoplasmic shuttling of YAP/TAZ and their implications for cancer, and discuss how the new insights about this process may pave the way for novel therapeutic strategies.

**Abbreviations**
CAFs: cancer‐associated fibroblasts
GPCR: G protein‐coupled receptors
HCC: hepatocellular carcinoma
LATS1 and LATS2: large tumor suppressor 1 and 2
NES: nuclear export signal
NLS: nuclear localization signal
TAZ: transcriptional co‐activator with PDZ‐binding motif
YAP: Yes‐associated protein
Yki: Yorkie

## 1 Introduction

The transcriptional co‐factors Yes‐associated protein (YAP) and transcriptional co‐activator with PDZ‐binding motif (TAZ), the main effectors of the Hippo signal transduction pathway, are emerging as pivotal determinants of malignancy in human cancer (Harvey *et al*., 2013; Zanconato *et al*., 2016a 2016b). In addition, mounting evidence suggests that they play important roles in chemotherapeutic drug resistance and have significant impact on patient prognosis (Zhao and Yang, 2015). For instance, high levels of YAP and TAZ are observed in many human liver tumors (Han *et al*., 2014). Furthermore, liver‐specific YAP overexpression in transgenic mice leads to hepatocellular carcinoma (HCC) development, suggesting that YAP is a key driver of tumorigenesis in this type of cancer (Dong *et al*., 2007). Moreover, YAP was shown to mediate cisplatin resistance in HCC (Mao *et al*., 2014). Similarly, tumors with high TAZ levels are more invasive and metastatic and therefore difficult to treat. For example, TAZ contributes to Taxol resistance in breast cancer cells (Lai *et al*., 2011). Furthermore, it was shown that YAP/TAZ activity contributes to lung tumor progression and metastasis (Lau *et al*., 2014). More broadly, high YAP and/or TAZ expression and/or aberrant nuclear localization are correlated with poor prognosis in many cancers, such as pancreatic adenocarcinoma, endometrial carcinoma, melanoma, squamous cell carcinoma of the skin, Kaposi's sarcoma, colorectal cancer, gastric cancer, head and neck squamous cell carcinoma, ovarian cancer, urothelial carcinoma of the bladder, and esophageal squamous cell carcinoma (Zanconato *et al*., 2016a 2016b). All of these imply that overexpression and hyperactivation of YAP and TAZ favor tumorigenesis. Intriguingly, YAP may also act as a tumor suppressor in specific circumstances (Levy *et al*., 2007; Strano *et al*., 2005).

Despite the emerging importance of YAP and TAZ in cancer, the exact mechanisms driving their activation in human tumors still remain to be fully resolved. At the genomic level, Hippo pathway genes, including *YAP/TAZ*, are rarely mutated in cancer, with only a few exceptions in specific tumors (Chen *et al*., 2015; Wang *et al*., 2018). This suggests that additional and diverse mechanisms must control YAP/TAZ dysregulation in cancer; these might include epigenetic alterations, post‐translational modifications, crosstalk with various other pathways, and aberrant subcellular localization.

YAP/TAZ regulation is multilayered and involves numerous mechanisms and factors. Thus, a wide range of inputs including cell density, cell polarity, mechanical stress, ligands of G protein‐coupled receptors (GPCRs), and cellular energy status (Piccolo *et al*., 2014), have all been shown to regulate YAP/TAZ. In particular, the nucleo‐cytoplasmic distribution of YAP/TAZ is a key determinant of their activity and is a major target of their regulation by upstream components of the Hippo pathway. More specifically, the Hippo signaling pathway consists of a large network of proteins, the core of which is a conserved kinase cascade that limits tissue growth by promoting phosphorylation and inhibition of YAP and TAZ, or their orthologue Yorkie (Yki) in *Drosophila* (Huang *et al*., 2005; Lei *et al*., 2008; Zhao *et al*., 2007). Thus, when the Hippo pathway is inhibited, YAP/TAZ/Yki gain activity. Subsequently, the nuclear abundance of YAP/TAZ/Yki increases. Nuclear localization is crucial for the functionality of YAP/TAZ/Yki as transcriptional coactivators. The abundance of YAP/TAZ in the nucleus can be modulated by a variety of signaling pathways. For example, the mevalonate and the glucocorticoid receptor signaling pathways regulate YAP nuclear accumulation in breast cancer (Sorrentino *et al*., 2014, 2017). Indeed, YAP/TAZ nuclear localization can serve as a tool to screen for novel upstream modulators of the Hippo pathway (Sorrentino *et al*., 2014, 2017). As mentioned above, elevated nuclear presence of YAP and TAZ can often be observed in a variety of human malignancies, including liver, lung, breast, skin, colon, and ovarian cancer (Harvey *et al*., 2013; Johnson and Halder, 2014; Piccolo *et al*., 2013).

Surprisingly, YAP/TAZ/Yki lack a canonical nuclear localization signal (NLS), and therefore, the machinery responsible for their nuclear accumulation is not obvious (Wang *et al*., 2016b). Recently, Gao *et al*. (2017) added another layer of complexity to the picture. By using super‐resolution microcopy, they observed that, within several human cell lines, YAP is mainly distributed in nuclear clusters. Interestingly, cell contact and mechanical pressure weakened YAP clustering and transcriptional activity (Gao *et al*., 2017). Thus, not only subcellular, but also subnuclear distribution might be critical in the regulation of YAP transcriptional activity.

All in all, it is of great interest to understand how the nucleo‐cytoplasmic shuttling of these proteins is maintained and regulated under normal conditions, and how it becomes deregulated in cancer. Several recent studies (Ege *et al*., 2018; Elosegui‐Artola *et al*., 2017; Kofler *et al*., 2018; Manning *et al*., 2018) have begun to unravel this mystery. Here, we review the current understanding and discuss how these new insights may be exploited therapeutically.

## 2 Then and now: Regulation of YAP/TAZ/Yki nucleo‐cytoplasmic shuttling by the Hippo pathway

According to the canonical view of the mammalian Hippo pathway, mammalian STE20‐like protein kinase 1 and 2, together with the adaptor protein Salvador homologue 1, phosphorylate and activate large tumor suppressor 1 and 2 (LATS1 and LATS2). Activated LATS1/2, together with the adaptor proteins MOB kinase activator 1A and 1B, in turn phosphorylate YAP and TAZ. It is generally accepted that phosphorylation by LATS1/2 leads to YAP/TAZ nuclear exclusion, cytoplasmic sequestration by 14‐3‐3 anchoring factors, and/or proteasomal degradation. In *Drosophila*, the subcellular localization of Yki is regulated similarly by phosphorylation by the LATS orthologue Warts and subsequent 14‐3‐3 binding (Dong *et al*., 2007; Yu and Guan, 2013; Zhao *et al*., 2007). In the nucleus, Yki and YAP/TAZ exert their biological effects by regulating gene transcription (Dong *et al*., 2007; Yu and Guan, 2013; Zhao *et al*., 2007). Thus, through regulating the subcellular localization of YAP/TAZ/Yki, the Hippo kinase cascade maintains temporal control of their activity (Yu and Guan, 2013). Accordingly, prevention of YAP/Yki phosphorylation affects their biological functions and specifically increases their growth‐promoting activity (Dong *et al*., 2007; Zhao *et al*., 2007). Since YAP and TAZ do not have sequence‐specific DNA‐binding capability, they depend on interaction with sequence‐specific transcription factors for their recruitment to target sequences in the chromatin. In particular, TEAD family transcription factors serve as such chromatin anchors and therefore play an essential role in YAP/TAZ‐dependent gene expression and cell growth stimulation (Zhao *et al*., 2008).

Several recent studies have expanded this canonical model. First, contrary to the above dogma, it was shown that LATS‐dependent phosphorylated YAP can be retained in the nucleus and is not necessarily exported to the cytoplasm (Wada *et al*., 2011). Based on these observations and additional experiments, it was concluded that although phosphorylation of YAP is required for its exclusion from the nucleus, it not sufficient by itself (Wada *et al*., 2011), contrary to the canonical model. Second, in a study by Dupont *et al*. (2011) establishing the connection between YAP/TAZ and mechanotransduction, they showed that YAP/TAZ are entrapped in the cytoplasm upon treatment with cytoskeleton inhibitors. However, more in‐depth analysis revealed that YAP/TAZ actually shuttle between the cytoplasm and the nucleus, rather than being statically tethered within one subcellular compartment. This was shown by treating cells with a combination of both cytoskeletal inhibitors and nuclear export inhibitors; under such combined treatment, the cells exhibited increased nuclear localization of YAP/TAZ compared to treatment with cytoskeletal inhibitors alone, implying that they can shuttle into the nucleus and back even when the cytoskeleton is disrupted.

Challenging the existing model even further, Wang *et al*. (2016a) identified a biphasic YAP localization pattern in intestinal epithelial cells in response to mitogenic GPCR agonists. Specifically, they observed that YAP first translocates to the cytoplasm, and this is then followed by subsequent re‐entry into the nucleus, eventually driving changes in gene expression. The observations reported by these researchers are provocative for several reasons. First, whereas treatment with GPCR agonists or serum growth factors caused nuclear accumulation of YAP in some cell lines, it actually resulted in cytoplasmic sequestration in others. Thus, it seems that the same signal can either activate or inactivate YAP, depending on cellular context. Second, their findings imply that transient nuclear exit of YAP during the first phase is necessary for its nuclear re‐entry and activation. More specifically, exposure to leptomycin B, a nuclear export inhibitor, prevented the nuclear exit of YAP upon exposure to a GPCR agonist. Counterintuitively, it also blocked the expression of YAP‐regulated genes, despite the continued presence of YAP in the nucleus. Altogether, this suggests that dynamic nucleo‐cytoplasmic shuttling of YAP is important for its function, implying that YAP needs to be localized in the right place at the right time.

In addition, four new studies (Ege *et al*., 2018; Elosegui‐Artola *et al*., 2017; Kofler *et al*., 2018; Manning *et al*., 2018) thoroughly investigated mechanistic aspects of the regulation of YAP/TAZ/Yki nucleo‐cytoplasmic translocation. Three of these (Ege *et al*., 2018; Elosegui‐Artola *et al*., 2017; Manning *et al*., 2018) employed a similar approach, in which fluorescently labeled YAP/Yki was monitored by advanced microscopy tools and live imaging, to infer subcellular dynamics upon various treatments. Importantly, all three studies concluded that the majority of YAP/TAZ/Yki molecules rapidly and dynamically traffic between the cytoplasm and the nucleus.

Beyond the overall similar conclusions, each group also presented distinct mechanistic findings. Ege *et al*. (2018) compared YAP nuclear export and import rates in normal vs. cancer‐associated fibroblasts (CAFs) by using exogenously expressed YFP‐YAP protein and combining photobleaching with mathematical modeling. They concluded that nuclear export, accelerated by LATS‐mediated phosphorylation, is the key determinant of YAP localization. They identified exportin 1 (XPO1) as the exportin used by YAP to exit the nucleus. Surprisingly, dephosphorylation was not sufficient for YAP activation, and tyrosine phosphorylation by Src‐family kinases and cues from the cytoskeleton were necessary for effective YAP transcriptional activity.

Elosegui‐Artola *et al*. (2017) uncovered a novel mechanosensing mechanism directly converting force into nuclear import. They found that nuclear flattening, caused by mechanical force, leads to increased nuclear entry of YAP (and, potentially, other proteins) due to decreased mechanical restriction of molecular transport through nuclear pores. More specifically, fluorescence recovery after photobleaching and atomic force microscopy were used to follow the localization of overexpressed GFP‐YAP in mouse fibroblasts upon mechanical stress‐associated treatments. Interestingly, application of force to the nucleus was sufficient to translocate YAP into the nucleus independently of surface rigidity, focal adhesions, actin cytoskeleton, cell–cell adhesion, or LATS and MST overexpression. This suggests that nucleoskeletal changes override other signals to govern YAP subcellular localization. Interestingly, this study concluded that the nuclear import rate determines YAP location and activity.

Manning *et al*. (2018) explored Hippo pathway dynamics, focusing on the fly orthologue of YAP/TAZ, Yki. They endogenously tagged Yki with YFP and followed its dynamics in both larval wing and pupal notum. Intriguingly, they found that cell populations within the larval wing displayed different rates of Yki nucleo‐cytoplasmic shuttling. These differences suggest that regulation of YAP/TAZ/Yki nuclear localization is cell‐type‐specific even within the same tissue. Similar observations were also described in an earlier study on mammalian YAP localization in the developing lung (Mahoney *et al*., 2014). Furthermore, Yki localization was found to be cell cycle‐dependent, in that Yki was primarily cytoplasmic during interphase but chromatin‐bound in mitosis. By using *Warts* mutants, Manning *et al*. could show that Yki nuclear import rates were Hippo pathway dependent, similar to the findings of Elosegui‐Artola *et al*.

The aforementioned studies mainly focused on YAP/Yki localization. In parallel, a recent study by Kofler *et al*. (2018) elucidated the molecular requirements underlying TAZ nucleo‐cytoplasmic shuttling. By using diffusion‐limited TAZ constructs and inducible nuclear influx and efflux systems in pig cells, they demonstrated that TAZ localization is highly regulated. Specifically, they found that RhoA stimulation increased the nuclear import of TAZ. RhoA, a member of the Ras‐related family of GTPases and regulator of cytoskeleton dynamics (Hall, 1998; Ridley and Hall, 1992), has already been linked previously to TAZ/YAP activation (Ege *et al*., 2018; Elosegui‐Artola *et al*., 2017). Interestingly, at variance with the current dogma that YAP/TAZ lack nuclear import signals, Kofler *et al*. identified a noncanonical NLS in TAZ. In addition, they also identified a novel nuclear export signal (NES); both the NLS and the NES, identified in pig cells, are conserved also in human TAZ and YAP. The TAZ NLS represents a new class of import motifs, necessary and sufficient for efficient nuclear uptake, whereas the TAZ NES overlaps with the binding site for TEAD proteins. TEAD binding was previously shown to modulate YAP/TAZ nuclear localization (Chan *et al*., 2009; Ege *et al*., 2018; Lin *et al*., 2017); the recent findings provide an interesting explanation for this observation, namely that TEAD binding can mask the NES and consequently dampen nuclear export.

**Figure 1:**
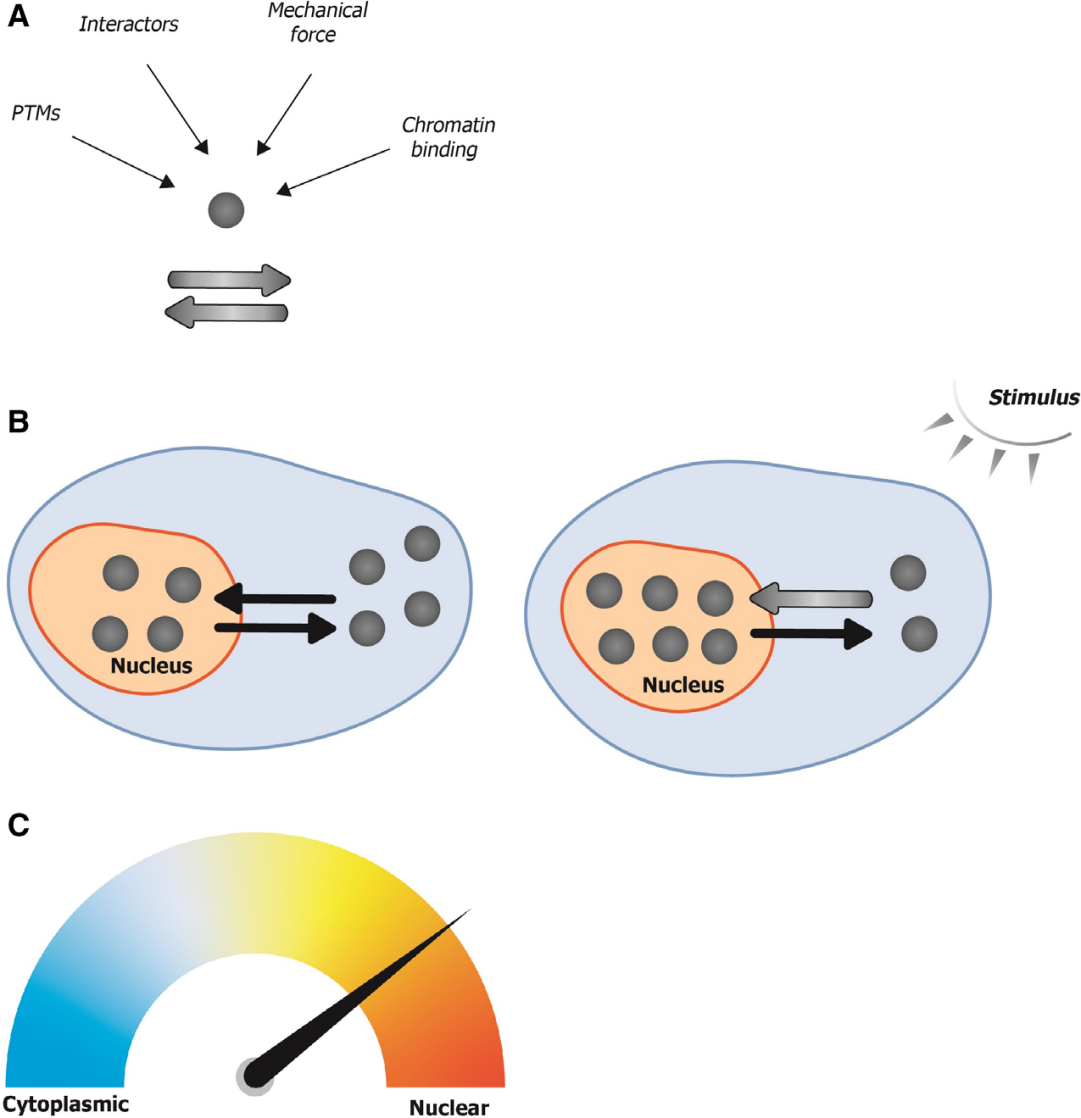
The balance between export and import rates dictates YAP/TAZ/Yki subcellular localization, which is a continuous and dynamic process. (A) At the single protein level, YAP/TAZ/Yki can be subject to several modes of regulation, such as post‐translational modifications (PTMs), interactions with specific proteins, binding to chromatin, and/or mechanical forces that alter nuclear pore permeability. The sum of these dictates the rates of YAP/TAZ/Yki nuclear export/import. Importantly, this nucleo‐cytoplasmic shuttling is continuously ongoing. (B) At the cellular level, the constant shuttling results in an equilibrium, making it appear as though some of the molecules reside stably in the nucleus while others remain cytoplasmic. When an activating stimulus is delivered to the cell, the balance between export and import is shifted, such that more molecules eventually end up in the nucleus. Gray circles represent single YAP/TAZ/Yki molecules. Black arrows represent the direction of translocation. A thicker gray arrow indicates a higher rate. (C) At the population level, YAP/TAZ/Yki nucleo‐cytoplasmic localization is not a binary state, but rather a range of states. The relative change between states is cell‐type‐ and/or stimulus‐dependent.

Together, these recent studies call for reevaluation of the canonical model of YAP/TAZ/Yki regulation. In agreement with previous models, YAP/TAZ/Yki transcriptional impact is primarily controlled by subcellular localization. However, a revised model would suggest that YAP/TAZ/Yki continually shuttle between the nucleus and the cytoplasm and are maintained at steady state by a balance of nuclear export and import rates. At the single molecule level, diverse modes of regulation, such as combinations of passive influx by controlling nuclear pore permeability with active regulation by protein modifications (like LATS/Warts serine phosphorylation or Src‐family kinases tyrosine phosphorylation) and interactions with specific binding partners (TEAD or 14‐3‐3), affect nuclear export and/or import rates (Fig. 1A). The outcome of this, at the cellular level, is that changes in the number of YAP/TAZ/Yki molecules in the nucleus lead to changes in transcriptional activation of their target genes (Fig. 1B). Thus, YAP/TAZ/Yki nucleo‐cytoplasmic shuttling is not a binary state, as suggested by classical models, but rather a snapshot of a range of continuous nuclear and cytoplasmic shuttling dynamics (Fig. 1C).

The complex subcellular regulation of YAP/TAZ/Yki underscores the need for their tight regulation in cell‐ and condition‐specific contexts. A combination of multiple points of regulation might allow a more refined response to different stimuli, which can result in a broad spectrum of nuclear to cytoplasmic YAP/TAZ/Yki ratios. Finally, the revised model suggests the existence of cell‐to‐cell variability with regard to YAP/TAZ/Yki subcellular localization, which might contribute to greater robustness at the population level.

## 3 Future perspectives

Several questions remain open. First, only a few exportins and importins have been shown so far to regulate directly YAP/TAZ/Yki nucleo‐cytoplasmic translocation (Ege *et al*., 2018; Wang *et al*., 2016b). It will thus be interesting to identify additional modulators of this process. Second, the apparent contradictions between some of the conclusions of the studies discussed above underline the importance of characterizing more comprehensively cell‐type‐ and condition‐specific YAP/TAZ/Yki subcellular dynamics. Furthermore, since YAP/TAZ display increased nuclear abundance in a variety of tumors (Zanconato *et al*., 2016b), it will be of great interest to elucidate the mechanisms responsible for their deregulated translocation in cancer cells.

Importantly, direct pharmacological inhibition of YAP/TAZ activity remains a clinical challenge. Current approaches toward meeting this challenge are reviewed in Guo and Teng (2015); Nakatani *et al*. (2016); Zanconato *et al*. (2016a, 2016b). For instance, a major effort is made for interfering with YAP/TAZ‐TEAD complexes. However, directly targeting YAP and TAZ may result in serious side effects, as YAP/TAZ are important for tissue homeostasis under physiological conditions. Thus, YAP/TAZ inhibition should preferably be targeted in a tissue‐specific and/or transient manner.

Inhibition of YAP/TAZ nuclear localization might serve as a potential therapeutic strategy, especially in view of the new insights gained from the aforementioned publications. One obvious approach might be to directly inhibit the nuclear export protein CRM1/XPO1. Indeed, this already represents a therapeutic promise in several types of cancer, and at least two such inhibitors are presently in clinical trials (Muqbil *et al*., 2018; Sun *et al*., 2016; Tai *et al*., 2014). Of course, these inhibitors will affect the localization of many additional proteins, but tumors with hyperactive nuclear YAP/TAZ may particularly be affected and may therefore be considered as prioritized candidates for such treatment. Furthermore, an interesting approach may be to target YAP/TAZ in the tumor microenvironment, especially in CAFs, in which YAP was shown to be activated and required for the tumor‐supportive functions of these stromal cells (Calvo *et al*., 2013). As shown by Ege *et al*. (2018), targeting actin or Src‐family kinases increases the rate of YAP export in CAFs. Consequently, YAP may become less nuclear and hence less active in the treated CAFs, thereby attenuating the contribution of the CAFs to tumor growth and therapy resistance (Chen and Song, 2019). YAP/TAZ nuclear accumulation may also be suppressed indirectly, by drugs that target signaling pathways responsible for increased nuclear translocation of YAP/TAZ. For example, one may consider in that regard inhibition of the mevalonate or the glucocorticoid receptor pathways (Sorrentino *et al*., 2014, 2017), for which there are already numerous FDA‐approved drugs. Lastly, as the above studies strengthen the link between the cytoskeleton and YAP/TAZ subcellular localization, existing cytoskeleton‐modulating drugs may be evaluated as YAP/TAZ‐targeted therapies (Piccolo *et al*., 2013); this is supported by the findings that inhibition of Rho or the actin cytoskeleton attenuates YAP/TAZ nuclear localization and transcriptional activity (Dupont *et al*., 2011; Ege *et al*., 2018; Piccolo *et al*., 2013; Wada *et al*., 2011).

Hopefully, answers to these questions will shed more light on the involvement of the Hippo pathway in tumorigenesis and open new directions for cancer therapy.

## Conflict of interest

The authors declare no conflict of interest.
